# Encoding arbitrary phase profiles to 2D diffraction orders with controllable polarization states

**DOI:** 10.1515/nanoph-2022-0707

**Published:** 2023-01-03

**Authors:** Ruizhe Zhao, Xin Li, Guangzhou Geng, Xiaowei Li, Junjie Li, Yongtian Wang, Lingling Huang

**Affiliations:** Beijing Engineering Research Center of Mixed Reality and Advanced Display, Key Laboratory of Photoelectronic Imaging Technology and System of Ministry of Education of China, School of Optics and Photonics, Beijing Institute of Technology, Beijing 100081, China; Beijing National Laboratory for Condensed Matter Physics, Institute of Physics, The Chinese Academy of Sciences, Beijing 100191, China; Laser Micro/Nano-Fabrication Laboratory, School of Mechanical Engineering, Beijing Institute of Technology, Beijing 100081, China

**Keywords:** diffraction order modulation, double-phase method, metasurface

## Abstract

Generating 2D diffraction orders with uniform or tailored intensity distribution is highly desired for various applications including depth perception, parallel laser fabrication and optical tweezer. However, previous strategies lack the abilities to tailor multiple parameters of output light in different diffraction orders simultaneously. While such ability plays an important role in achieving various different functionalities parallelly. Here, we demonstrate a method for encoding arbitrary phase profiles to different diffraction orders with controllable polarization states by applying double-phase method into elaborately designed metasurface. Sixteen independent holograms that generated by GS algorithm are successfully encoded into 4 × 4 uniformly distributed diffraction orders. Hence, the predefined holographic images can be observed at the Fourier plane. Meanwhile, the corresponding polarization states of different orders are manipulated according to their Fourier coefficients. For verifying the polarization state of each holographic image, we calculate the Stokes parameter of each order from measured intensity distributions in the experiment. The proposed method provides an effective way to tailor multiple properties of output diffraction orders. Meanwhile, it may promote the realization of achieving various functionalities parallelly such as spectral-polarization imaging or phase-polarization detection and enhance the capabilities of optical communication systems.

## 1 Introduction

Diffraction gratings provide an effective approach to manipulate the propagation direction or energy distribution of diffraction orders by adjusting the topography, grating period as well as duty cycle. They are crucial optical elements for various applications including optical communication, spectrometer, polarimeter and so on [1–3]. For obtaining 1D or 2D diffraction orders with uniform or tailored amplitude distributions, Dammann gratings provide a flexible approach to realize such parallel beam modulation. In each period of Dammann gratings, the phase profile can be optimized based on different optimization methods (genetic algorithm, simulated annealing algorithm or gradient descent optimization) with the assistant of predefining property evaluation functions [4–7]. The generations of multiple diffraction orders with high uniformity are highly desired for achieving depth perception, parallel laser fabrication and optical tweezer [8–10]. Furthermore, generalized phase functions including lens factor, spiral phase profile, axicon phase profile and cube phase profile can be encoded to the Dammann gratings. Therefore, multiple focal points [11], vortex beams array [12–14], Bessel beams array [15, 16], as well as Airy beams array [17, 18], with high uniformity can be realized. In these demonstrated strategies, the calculated phase profiles usually encoded to the same polarization channel of single device which lead to all the diffraction orders exhibit homogeneous polarization distribution. Meanwhile, the phase profiles that encoded to different diffraction orders are always identical which hinder such gratings to achieve various different functionalities parallelly.

In recent years, the ultrathin metasurfaces that composed of artificially designed meta-atoms have represented unprecedented abilities for tailoring multiple fundamental properties of light such as amplitude [19, 20], phase [21, 22], polarization [23–27], orbital angular momentum (OAM) [28, 29], etc. Such powerful functionalities are primarily attributed to the various design freedoms of composed meta-atoms as well as different wavefront modulation mechanisms. Especially, the multi-dimensional wavefront modulation empowered by metasurfaces in favour of realizing beam shaping and diffraction orders modulations. Dielectric metasurface with the capability of generating full-space cloud of Random points is demonstrated which pave the way to realize depth perception-related applications in compact optical systems [30]. More than 4044 random spots are observed with the lightweight and flexible designed metasurface. Based on the reciprocal process of the propagating beams, a metasurface chip is demonstrated for diffracting the incident beam into five beams with predefined directions and identical polarization states for cold atom trapping [31]. Such design strategy provides an alternative way to replace the conventional bulky optical elements used to produce a cold atomic ensemble. Furthermore, arbitrary and parallel polarization responses can be implemented to different output diffraction orders based on matrix Fourier optics method which leading to the realization of a compact full-Stokes polarization camera [32]. In addition, a supercell metasurface is proposed for achieving multiple independent optical functionalities at arbitrary large deflection angles with high efficiency by considering the non-local interactions due to optical coupling between neighbor meta-atoms [33]. Based on the versatile platform provided by the metasurfaces, some methods have demonstrated for tailoring the amplitude, the encoded phase profiles or polarization states of different diffraction orders. Nevertheless, the simultaneous modulations of multiple parameters of output diffraction orders are seldom investigated [34]. The ignored part may promote the advancement of achieving various different functionalities parallelly within single-layer metasurfaces.

Here, we demonstrate a method for encoding 4 × 4 independent holograms to different diffraction orders with controllable polarization states based on double-phase method with the assistance of an elaborated designed dielectric metasurface. Hence, different holographic images are successfully obtained at the predefined positions. The polarization states of each diffraction orders are determined by their Fourier coefficients. In the experiment, we calculate the Stokes parameter of different diffraction orders based on the captured intensity distribution under different configurations of a polarizer and a quarter waveplate. The calculated result is consistent with the theoretical expectation and successfully verify the polarization states of different orders. The demonstrated method provides an effective way to modulate multiple properties of output diffraction orders simultaneously. It may also promote the realization of various functionalities parallelly such as spectral-polarization imaging or phase-polarization detection and enhance the capabilities of optical communication systems.

## 2 Results

**Figure 1:**
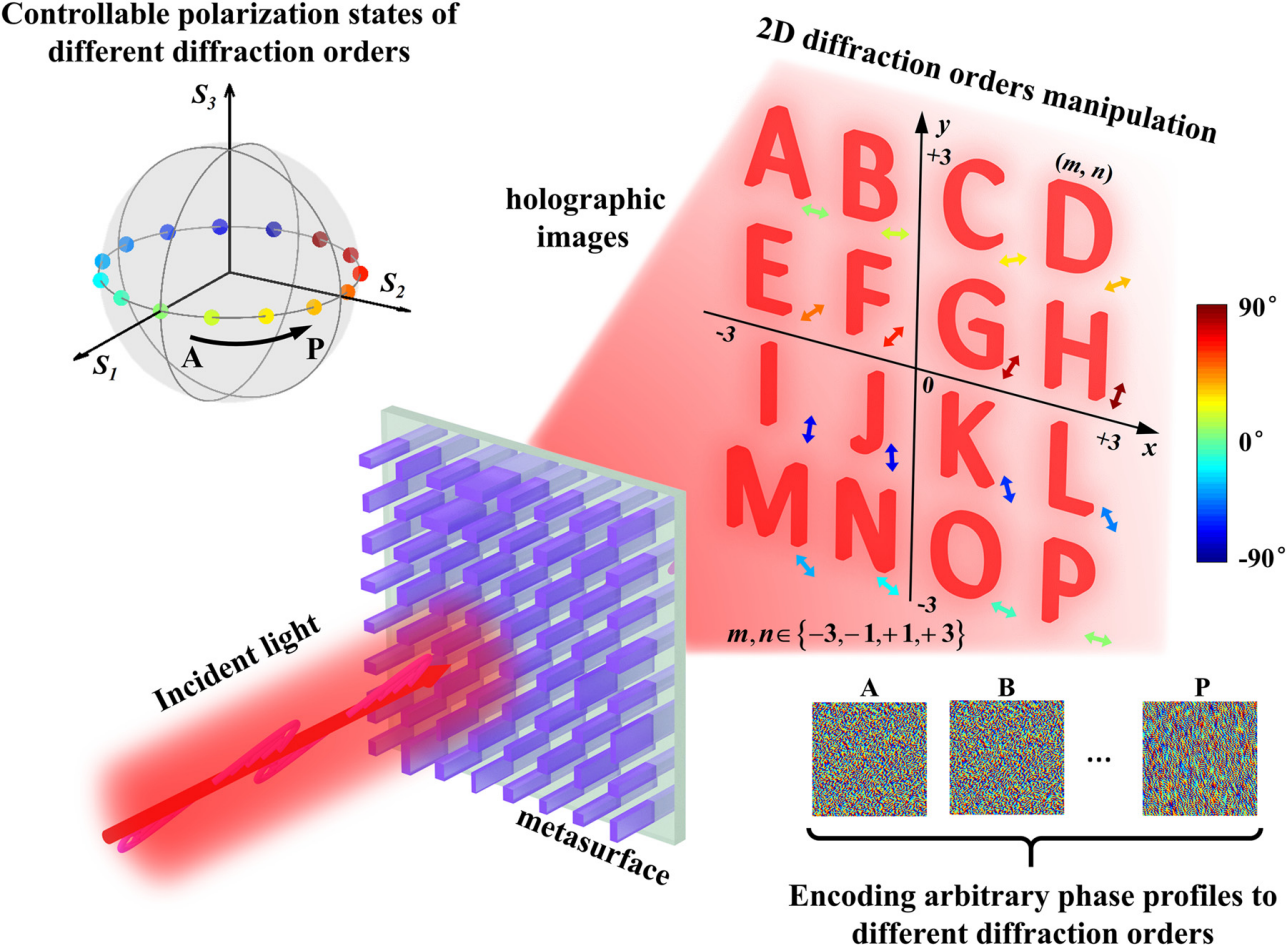
Schematic illustration of a dielectric metasurface with the capability of modulating multiple parameters of 2D diffraction orders simultaneously. Sixteen independent holograms are encoded to 4 × 4 uniformly distributed diffraction orders with controllable polarization states. All the holographic images exhibit linearly polarized states with different orientations that represented by the arrows with different colors. Meanwhile, the polarization states are also represented by the colorful spheres that located at the equator of the Poincaré sphere with equal intervals.

The schematic illustration of our demonstrated method for encoding arbitrary phase profiles to different diffraction orders with controllable polarization states is depicted in Figure 1. Firstly, sixteen capitalized alphabets (from A to P) are chosen as the original images and the corresponding holograms are calculated by traditional GS algorithm, separately. Then, we successfully encode such independent holograms to 4 × 4 uniformly distributed diffraction orders based on double-phase method with an elaborated designed dielectric metasurface. Hence, the predefined sixteen holographic images can be obtained at the Fourier plane. The polarization states of the acquired holographic images are represented by the colourful spheres that located at the equator of the Poincaré sphere with equal intervals. All the holographic images exhibit linearly polarized states with gradually changed orientation directions. Noted that the tailored polarization states are not limited to linear polarization states in our scheme. Circular and elliptical polarization states are also effective.

The transmission function of a grating with 2D output diffraction orders can be expressed as follow:

(1) $$\left\{\begin{array}{c} T_{h\text{\_}x}=\overset{\infty }{\underset{m=-\infty }{\sum }}\overset{\infty }{\underset{n=-\infty }{\sum }}c_{mn\text{\_}x}\exp\left[\text{i}\left(m\frac{2\pi }{D_{x}}x+n\frac{2\pi }{D_{y}}y\right)\right]\times \exp\left(\text{i}\varphi_{\text{holo}}^{mn}\right)\;\\ T_{h\text{\_}y}=\overset{\infty }{\underset{m=-\infty }{\sum }}\overset{\infty }{\underset{n=-\infty }{\sum }}c_{mn\text{\_}y}\exp\left[\text{i}\left(m\frac{2\pi }{D_{x}}x+n\frac{2\pi }{D_{y}}y\right)\right]\times \exp\left(\text{i}\varphi_{\text{holo}}^{mn}\right)\; \end{array}\right.$$

where *D*_*x*_ and *D*_*y*_ indicate the grating period in *x*- and *y*-direction and set as 9 μm in our scheme. The suitable super-period is selected in favour of capturing the output 4 × 4 uniformly distributed diffraction orders by using an objective lens with moderate numerical aperture. The *m* and *n* are used to represent the diffraction order (*m*, *n*) and set as −3, −1, +1 and +3 in our scheme. The hologram $\varphi_{\text{holo}}^{mn}$ refers the encoded phase profile into (*m*, *n*) order. Meanwhile, the *c*_*mn*_*x*_ and *c*_*mn*_*y*_ are the Fourier coefficients of the (*m*, *n*) order which can be represented by a Jones vector ${\left[c_{mn\text{\_}x},c_{mn\text{\_}y}\right]}^{T}$ . Hence, the polarization state of each diffraction order is completely determined by its Fourier coefficients. For the convenience of experiment verification, the polarization state of each order is set as linear polarization state with specific orientation angle which can be represented by ${\left[\cos\left(\theta_{mn}\right),\sin\left(\theta_{mn}\right)\right]}^{T}$ (*θ*_*mn*_ is in the range of –*π*/2 to *π*/2 with equal interval). Arbitrary polarization manipulation is commonly realized by superimposing two orthogonal polarization components with tailored amplitude ratio and phase delay. Such superimposing process is still needed for manipulating the polarization state of each diffraction order and can be realized by encoding the calculated complex amplitude distribution *T*_*h*_*x*_ and *T*_*h*_*y*_ to the orthogonal polarization channels of a single device (more details are provided in Section A in our Supporting Information).

Metasurfaces provide a versatile platform for manipulating the wavefront of output light and have the capabilities of achieving desired Jones matrices [35–37]. Especially, they exhibit superior performances in polarization modulation compare to traditional optical devices such as spatial light modulator (SLM) [38, 39]. Based on double-phase method, the complex amplitude modulations of two orthogonal polarization channels can be expressed by the sum of two unitary matrices *T*_1_ and *T*_2_ [40]:

(2) $$\begin{array}{ll} T & =\left[\begin{array}{cc} a_{xx}{\text{e}}^{\text{i}\varphi_{xx}} & 0\\ 0 & a_{yy}{\text{e}}^{\text{i}\varphi_{yy}} \end{array}\right]\\ & =\frac{1}{2}\left[\begin{array}{cc} {\text{e}}^{\text{i}\varphi_{xx}^{1}} & 0\\ 0 & {\text{e}}^{\text{i}\varphi_{yy}^{1}} \end{array}\right]+\frac{1}{2}\left[\begin{array}{cc} {\text{e}}^{\text{i}\varphi_{xx}^{2}} & 0\\ 0 & {\text{e}}^{\text{i}\varphi_{yy}^{2}} \end{array}\right] \end{array}$$

**Figure 2:**
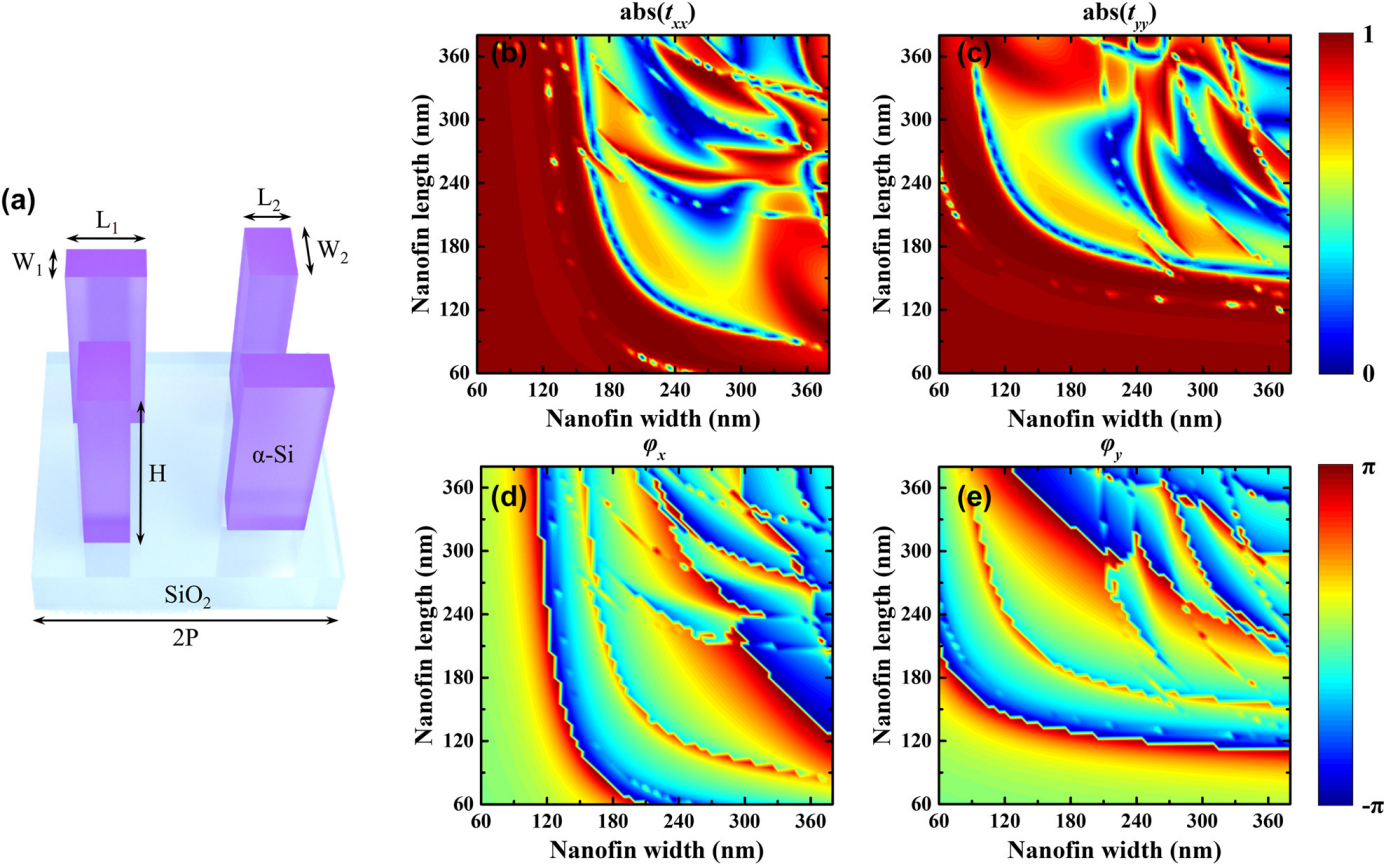
Simulated results of single nanofin. (a) The illustration of the supercell to constitute the demonstrated metasurface. Each supercell consists of 2 × 2 *α*-Si nanofins that set on a quartz substrate. The height of naonfin is 600 nm. And the period of the supercell is 900 nm. (b)–(e) Simulated amplitude (abs(*t*_*xx*_), abs(*t*_*yy*_)) and phase (*φ*_*x*_, *φ*_*y*_) of the transmission coefficients *t*_*xx*_ and *t*_*yy*_ by sweeping the length *L* and width *W* of single nanofin in the range of 60 nm–380 nm with an interval of 5 nm.

In Eq. (2), the $\varphi_{\mathrm{ii}}^{1}=\varphi_{ii}+\cos^{-1}\left(a_{ii}\right)$ and $\varphi_{\mathrm{ii}}^{2}=\varphi_{ii}-\cos^{-1}\left(a_{ii}\right)$ (*ii* = *xx* or *yy*) represent the phase items of *t*_*xx*_ and *t*_*yy*_ polarization channels in *T*_1_ and *T*_2_. Meanwhile, the diagonal and unitary matrix *T*_1_ and *T*_2_ can be easily realized by the lossless nanofin. As shown in Figure 2(a), we adopt the 2 × 2 amorphous silicon (*α*-Si) nanofins that set on a quartz substrate as the supercell to constitute our designed metasurface. Due to the form birefringence phenomenon of single *α*-Si nanofin, arbitrary phase delays can be imposed on the orthogonal polarization channels if the size of nanofin can be chosen freely. Hence, a 2D parameter optimization is carried out based on rigorous coupled wave analysis (RCWA) method by sweeping the length *L* and width *W* in the range of 60 nm–380 nm with an interval of 5 nm. In the optimization, the working wavelength is fixed at 800 nm and the corresponding refractive index of amorphous silicon and substrate are set as *n*_Si_ = 3.802 and *n*_sub_ = 1.5, respectively. The height *H* of naonfin is chosen as 600 nm in order to guarantee the imposed phase delays on orthogonal polarization channels can cover 0 to 2*π*. And the period *P* is set as 450 nm for alleviating neighbour coupling effect. The amplitude (abs(*t*_*xx*_), abs(*t*_*yy*_)) and phase (*φ*_*x*_, *φ*_*y*_) of the simulated transmission coefficients *t*_*xx*_ and *t*_*yy*_ are shown in Figure 2(b)–(d). In each supercell, the sizes of the nanofins that located at diagonal and anti-diagonal positions are determined by guaranteeing the minimum of the error $\varepsilon =abs\left(t_{xx}-\exp\left(i\varphi_{xx}^{ii}\right)\right)+abs\left(t_{yy}-\exp\left(\right.\;i\varphi_{yy}^{ii}\left.\right)\right)$ (*ii* = 1, 2). Therefore, the demonstrated supercell can be utilized to encode the normalized complex amplitude distributions *T*_*h*_*x*_ and *T*_*h*_*y*_ to orthogonal polarization channels of the metasurface, simultaneously.

**Figure 3:**
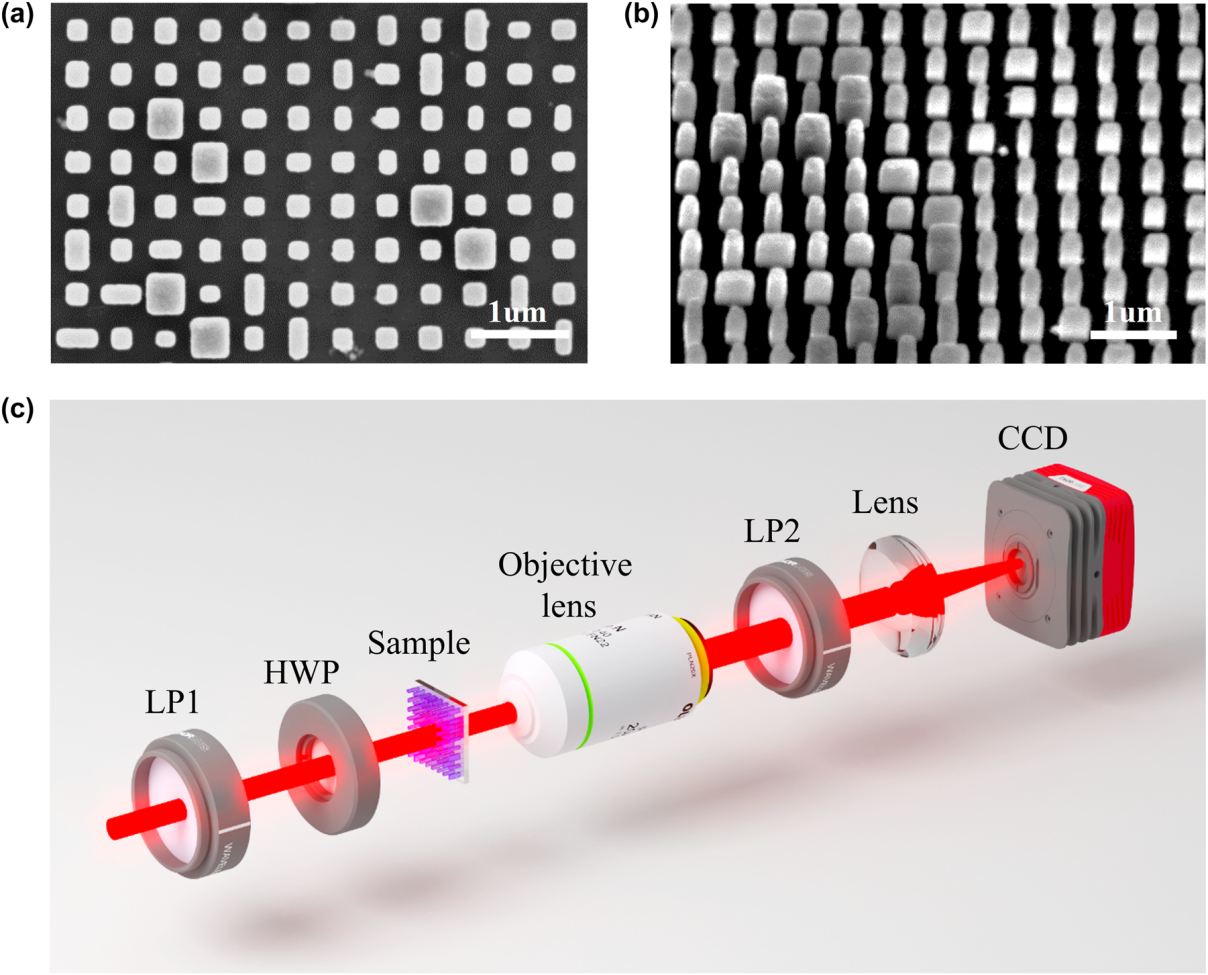
Sample fabrication and experimental setup. (a) and (b) The scanning electron microscopy images of our fabricated metasurface from top and side view. The metasurface is composed of 1000 × 1000 supercells. (c) The experimental setup for capturing the reconstructed holographic images at the Fourier plane.

For the purpose of experimental verification, we fabricate the demonstrated metasurface on top of a fused quartz substrate by using electron beam lithography (EBL) method. The scanning electron microscopy images of the fabricated metasurface from top view and side view are shown in Figure 3(a) and (b). The fabricated metausfrace consists of 1000 × 1000 super-cells and its total dimension is 900 μm × 900 μm. The experimental setup utilized for capturing the images that located at the Fourier plane is shown in Figure 3(c). A linear polarizer LP1 and a half-wave plate HWP are used together to manipulate the polarization state of incident light that illuminating on the metasurface. An objective lens with appropriate numerical aperture (40×/NA = 0.55) is utilized to collect the diffracted light from the metasurface. A charge coupled device camera (CCD) is placed at the back focal plane of a lens (*f* = 100 mm) in order to capture the reconstructed images. For measuring the Stokes parameters *S*_0_, *S*_1_ and *S*_2_ of the reconstructed images, another linear polarizer LP2 that used as a polarization analyzer is placed behind the metasurface. And the Stokes parameter *S*_3_ is obtained based on the configuration of LP2 as well as a quarter-wave plate (not shown in Figure 3(c)).

**Figure 4:**
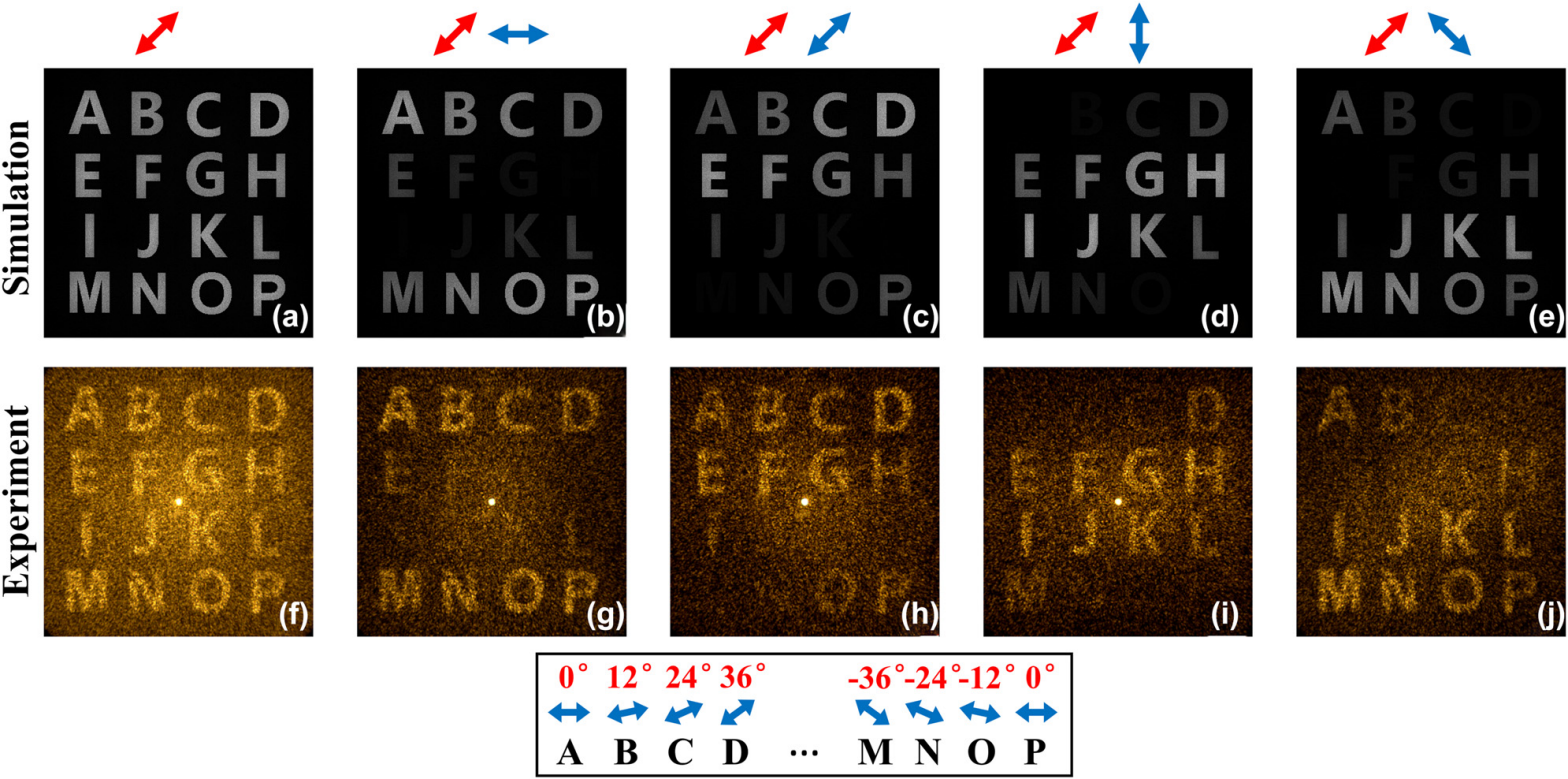
Simulated and experimental results of the reconstructed images. (a)–(j) Simulated and experimental results of the reconstructed holographic images in different diffraction orders that located at the Fourier plane. The red and blue arrows indicate the polarizations of incident and output light. The theoretical polarization states of the reconstructed images (from A to P) are represented in the bottom panel.

When 45° linearly polarized light illuminating on the fabricated metasurface, 4 × 4 uniformly distributed diffraction orders can be observed. For the diffraction order (*m*, *n*), it carries independent phase profile $\varphi_{\text{holo}}^{mn}$ with controllable polarization state. Thus, such functionality will enable the reconstruction of 4 × 4 holographic images with distinct vectorial features. The corresponding simulated and experimental results are illustrated in Figure 4(a)–(j). Sixteen capitalized alphabets (from A to P) are successfully captured at the predefined diffraction orders. Meanwhile, the polarization analyzer LP2 is utilized to distinguish the inhomogeneous polarization states. By successively rotating the transmission axis of LP2 to 0°, 45°, 90° and 135°, expected extinct phenomena are observed due to Malus’s law (more details are provided in video S1). Such phenomena are consistent with the simulated results. Noted that the multiple parameters of (0, 0) diffraction order can also be modulated. However, the generated hologram is not encoded to (0, 0) order in our current scheme. And we purposely manipulate the energy distribution in the desired diffraction orders while suppress all the unwanted orders. Therefore, the calculated zero-order diffraction is relatively weak. In the experiment, the zero-order diffraction as well as the background noises are slightly obvious. The zero-order without modulation is mainly caused by the incident light that transmits directly through the metasurface. Meanwhile, the fabrication error as well as the coupling between nanofins in each unit-cell also lead to the deviations of obatined complex amplitude distributions (*T*_*h*_*x*_ and *T*_*h*_*y*_) to the desired one. These reasons will cause the appearances of zero-order and the background noises.

**Video S1:** 

For determining the exact polarization states of the reconstructed images, we acquire the Stokes parameters at each pixel of the Fourier plane by measuring the intensity distributions under different configurations of polarization analyzers based on Eq. (3).

(3) $$\left\{\begin{array}{c} S_{0}=I_{0}+I_{90}\\ S_{1}=I_{0}-I_{90}\\ S_{2}=I_{45}-I_{135}\\ S_{3}=I_{\text{R}}-I_{\text{L}} \end{array}\right.$$

**Figure 5:**
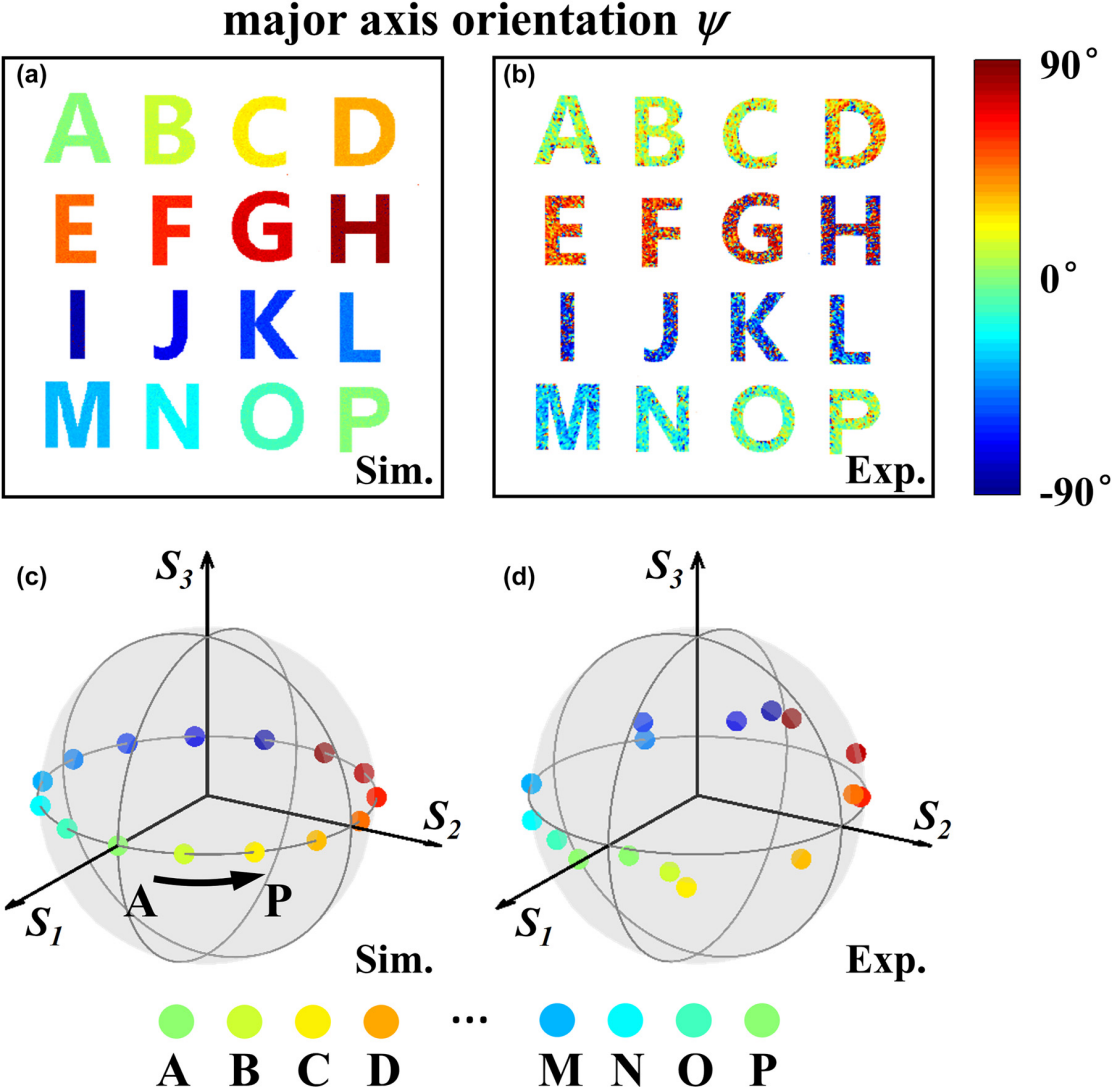
Polarization information of the reconstructed images. (a) and (b) Simulated and experimental results of the major axis orientation at the Fourier plane. We only concentrate on the results in the area of the reconstructed images. The polarization information of the background is set transparent. (c) and (d) Simulated and experimental results of the polarization states of the reconstructed holographic images in different diffraction orders. The polarization states of the images are represented by the spheres with different colors in a Poincaré sphere.

The intensity distributions *I*_0_, *I*_45_, *I*_90_ and *I*_135_ are captured by rotating the transmission axis of LP2 to specific orientation angle. Meanwhile, *I*_R_ and *I*_L_ refer the right-handedness and left-handedness circular polarization components. The simulated and measured major axis orientation *ψ* (*ψ* = tan^−1^(*S*_2_/*S*_1_)/2) of the polarization ellipse at each pixel of the Fourier plane are shown in Figure 5(a), (b). For the reconstructed images (from A to P), the corresponding orientations are gradually rotated in the range of –*π*/2 to *π*/2. Noted that we only concentrate on the results in the area of the reconstructed images. The polarization information of the background is set transparent. Furthermore, we represent the simulated and measured Stokes parameters of the sixteen reconstructed images on a Poincaré sphere as shown in Figure 5(c)–(d). For each image, the corresponding Stokes parameter is the average value of the Stokes parameter at each pixel of the image area. From the measured results, we can observe that the polarization states of majority reconstructed images are located around the equator of the Poincaré sphere which consist with the simulated results in acceptable deviation. In the experiment, the measured transmission efficiency of the fabricated metasurface is 21.08%. The relative low efficiency is mainly caused by the complex amplitude modulation of our scheme. This may be improved by manipulating the Fourier coefficients *c*_*mn*_*x*_ and *c*_*mn*_*y*_ with advanced optimized algorithms or deep learning method and realize the similar functionality based on a phase-only platform. Furthermore, some discussions about the maximum diffraction angle of our current scheme are provided in Section C in our Supporting Information. And the comparison between our demonstrate scheme with vectorial holography approaches [41–44] are provided in Section D in our Supporting Information.

## 3 Conclusions

In summary, we have demonstrated a method for simultaneously modulating multiple properties of output 2D diffraction orders based on a dielectric metasurface. We successfully encode arbitrary phase profiles to 4 × 4 uniformly distributed diffraction orders with controllable polarization states by tailoring the complex amplitude distributions of the orthogonal polarization channels. Therefore, sixteen holographic images located at predefined diffraction orders with distinct vectorial features are realized. Meanwhile, even more display channels with uniform or tailored amplitude can be achieved by utilizing diffraction orders. Stokes parameter method is adopted to verify the polarization states of the reconstructed images. The measured results are consistent with the simulated results in acceptable deviations.

For the purpose of alleviating the coupling issue and improving the performance of reconstructed images, inverse design method or suitable optimization approaches are preferred to straightly design the unit-cell with the capability of modulating the complex amplitude distribution of two orthogonal polarization channels of output light. But such method is very time consuming especially for metasurface with large arrays. While our demonstrated method here provides an effective method for modulating multiple properties of output 2D diffraction orders and prompt the advancements of realizing various optical functionalities parallelly. Furthermore, it may pave the way to enhance the capability of optical communication systems by realizing polarization modulated vortex beams with different OAMs in different orders.

## Supplementary Material

Supplementary Material Details
