# Supplementary material for: Encoding arbitrary phase profiles to 2D diffraction orders with controllable polarization states
Source: Nanophotonics. 2023 Jan 3;12(1):155–63. doi: 10.1515/nanoph-2022-0707 (PMC11501726; doi:10.1515/nanoph-2022-0707)
Supplement: Supplementary file 3 — Supplementary Material Details [file j_nanoph-2022-0707_suppl_001.docx]

**Supplementary Information**

Encoding arbitrary phase profiles to 2D diffraction orders with controllable polarization states

Ruizhe Zhao^1^, Xin Li^1^, Guangzhou Geng^2^, Xiaowei Li^3^, Junjie Li^2^, Yongtian Wang^1^, Lingling Huang1[[1]](#footnote-1)

1. *Beijing Engineering Research Center of Mixed Reality and Advanced Display, Key Laboratory of Photoelectronic Imaging Technology and System of Ministry of Education of China, School of Optics and Photonics, Beijing Institute of Technology, Beijing, 100081, China*
2. *Beijing National Laboratory for Condensed Matter Physics, Institute of Physics, The Chinese Academy of Sciences, Beijing, 100191, China*
3. *Laser Micro/Nano-Fabrication Laboratory, School of Mechanical Engineering, Beijing Institute of Technology, Beijing 100081, China*
4. **Generating diffraction orders with arbitrary polarization states.**

The polarization state of each diffraction order is completely determined by its Fourier coefficients *cmn_x* and *cmn_y*. For an arbitrary polarization state $|\lambda\rangle$, the corresponding Jones vector can be expressed as $|\lambda\rangle\text{= }\left[\cos\chi, e^{i\delta}\sin\chi\right]^{T}$ (-π ≤ *χ* ≤ π, -π ≤ *δ=**δ_y_* - *δ_x_* ≤ π) in linear polarization basis. Arbitrary polarization states with tailored azimuth and ellipticity angle can be generated in our scheme by manupulating the *cmn_x* and *cmn_y* of each diffraction order. For proving that our demonstrated scheme is still effective for generating diffraction orders with circular and elliptical polarization states, we carry out a simulation of a designed metasurface based on finite difference time domain (FDTD) method. The simulated metasurface is composed of 60×60 supercells (size: 54×54 μm^2^). When 45° linearly polarized light illuminating on the designed metasurface, two holographic images (a square and a circle pattern) can be obtained in (-1,-1) and (+1,+1) diffracttion orders with left-handedness circular polarization (LCP) as well as elliptical polarization states. The corresponding Fourier coefficients of different orders can be expressed in the form on Jone vectors ($\left[1, \text{i} \right]^{T}$ and $\left[\cos(\pi/5), e^{i(-\pi/4)}\sin(\pi/5) \right]^{T}$). The *D_x_* and *D_y_* in the simulation are set as 5.4 μm. The holograms ( and ) for the diffraction order of (-1, -1) and (+1,+1) are obtained based on traditional GS algorithm, respectively. The desired complex amplitude distribution *Th_x* and *Th_y* can be calculated by Eq. (1) and encoded to the designed metasurface based on the method that demonstrated in the maintext.

In the simulation, perfectly-matched-layer (PML) boundary conditions are set in the *x-, y-* and *z-*directions. A 2D monitor is located several wavelengths away from the simulated metasurface in order to extract the distribution of the electromagnetic field. Then, the extracted distribution of electromagnetic field can be utilized to calculate the field distribution at the Fouerier plane based on far-field projection in FDTD. The theoretical and simulated results of the reconstructed images under different polarization analyzers are shown in **Figure S1 (a)-(j).** Meanwhile, the polarization ellipses of the reconstruacted images are shown in **Figure S1 (l)-(m)**. The polarization ellipses are determined based on the the simulated *E_x_* and *E_y_* polarization component of the reconstrcuted images and agree well with the target polarization ellipses. Therefore, the simualted results shown in **Figure S1** successfully prove that our demonstrate method can encode arbitrary phase profiles to different diffraction orders with controllable polarization states. The azimuth and ellipticity angles of each diffraction order can be manipulated arbitrarily.

1. **The realization of amplitude-only and phase-only modulation.**

Our demonstrated method is based on the complex amplitude modulations of the two orthogonal polarization channels of the metasurface. By utilizing the double-phase method, the complex amplitude modulations of two orthogonal polarization channels can be expressed by the sum of two unitary matrices *T_1_* and *T_2_* as expressed by Eq. (2) in the maintext. Meanwhile, the diagonal and unitary matrix *T_1_* and *T_2_* can be easily realized by the lossless nanofin. The target phases and can be implemented by nanofins with specific sizes based on propagation phases. Furthermore, such decomposition is still effective when the matrix *T* contain amplitude-only or phase-only items.

For proving that our proposed method can achieve either amplitude or phase modulation, we design a metasurface (50×50 supercells) with the capability of printing a binary star pattern in the real-space (amplitude-only modulation) and reconstructing a holographic image at the Fourier plane (phase-only modulation) in *t_xx_* and *t_yy_* polarization channels, respectively. The corresponding simulated results are shown in **Figure S2**.

1. **The maximum diffraction angle of our current scheme.**

In our design, the grating period *D_x_* and *D_y_* in *x*- and *y*-direction are set as 9 μm in our scheme. And the corresponding diffraction angle *α_+3,+3_* of highest diffraction order (+3, +3) is 24.87°. In the experiment, an objective lens is utilized to collect all the diffracted light from the metasurface. The numerical aperture (*NA*) of the hologram can be expressed as follow (*ref. 25*):

$\frac{l}{N\times s}=\frac{\sin\alpha}{\lambda}$ (S1)

where *N* and *s* refer the number of pixels and pixel size of metasurface, respectively, and *l* indicates the distance from a point of the original image to its center in the Fourier plane. In the process of designing the hologram, we need to guarantee all the desired images located inside the circle that represent the observing region of the objetive lens with specific NA($NA=n\times\sin\alpha$) as illustrated in **Figure S3.**

Due to the period of the supercell is larger than the wavelength of incident light (*s* =2*P*=900 nm > *λ*), high order reconstructed images will appear in large diffraction angle. For such grating effect, the diffraction angle *β_q_* of high order reconstructed images in *x*- or *y*-direction can be expressed as follow (*ref. 42*):

$\beta_{q}=\arcsin\left(\frac{q\lambda}{\text{s}}\right) (q=\pm1, \pm2, ... , \pm Q)$ (S2)

Thus, the calculated diffraction angles of order *β_±1,0_*/*β_0,±1_* are (±62.73°, 0°)/(0°, ±62.73°). For avoiding overlap between desired images located in *β_0,0_* and adjacent *β_±1,0_*/*β_0,±1_* orders (high order reconstructed images), the largest diffraction angle *α*_max_=26.38° (under the condition *l*=500, *N*=1000) can be determined based on Eq. (S2) in our current design. The calculated *α*_max_ is moderate and slightly larger than the largest experimental obtained diffraction angle *α_+3,+3_*. Hence, for the diffraction order that the corresponding diffraction angle is below than *α*_max_, uniform intensity and desired polarization modulation can be realized.

1. **The comparison between our demonstrate scheme with vectorial holography approaches.**

Vectorial holography refers to the approaches which can reconstruct holographic images with inhomogeneous polarization distributions. Both the 2D diffraction order manipulation and the vectorial holography scheme need to encode calculated phase/complex amplitude distributions to specific polarization channels of designed metasurface. The approaches of realizing vectorial holography can be primary classified into three categories ((i) segmented vectorial metasurface (ii) interleaved vectorial metasurface (iii) non-interleaved vectorial metasurface) based on the configurations of metasurface (*ref. 45*). For the segmented/interleaved approaches, achieve 2D diffraction order engineering with limited diffraction orders are relatively convenient. The diffraction order engineering can be implemented by encoding the phase profile to specific polarization channel based on simultaneously tailoring the phase and polarization state of output light (*ref. 26, ref.27, ref. 42*). However, for manipulating large number diffraction orders, the performances and efficiencies of such schemes will degrade due to spatial multiplexing approaches. And non-interleaved metasurfaces are preferred to achieve 2D diffraction order manipulation with large diffraction orders. Non-interleaved metasurfaces (*ref. 25, ref. 41*) can generate vectorial holographic images by encoding desired phase profiles to orthogonal polarization channels of output light (e.g. *t_xx_* and *t_yx_* channel). The desired phase profiles are obtained through iterations under specific restriction (determined by the desired polarization distributions at the Fourier plane). Such schemes can be adopted to generate 2D diffraction orders with uniform or tailored amplitude distributions as well as controllable polarization states. But it may be difficult to encode generalized phase profiles to desired diffraction orders, simultaneously. Therefore, advanced design strategies or optimized algorithms are worth to be investigated to realize such functionality based on a phase-only platform. Vectorial holography based on metasufraces have been extensively studied in recent years. While, only a few schemes are proposed for simultaneously manipulating multiple parameters of output 2D diffraction orders. The differences between vectorial holography approaches and our demonstrated scheme for 2D diffraction orders modulation are summarized in **Table S1**.

**Reference：**

45. Q. Song, X. Liu, C.-W. Qiu, et al., "Vectorial metasurface holography," *Appl. Phys. Rev.*, vol. 9, no. 1, pp. 011311, 2022.

**
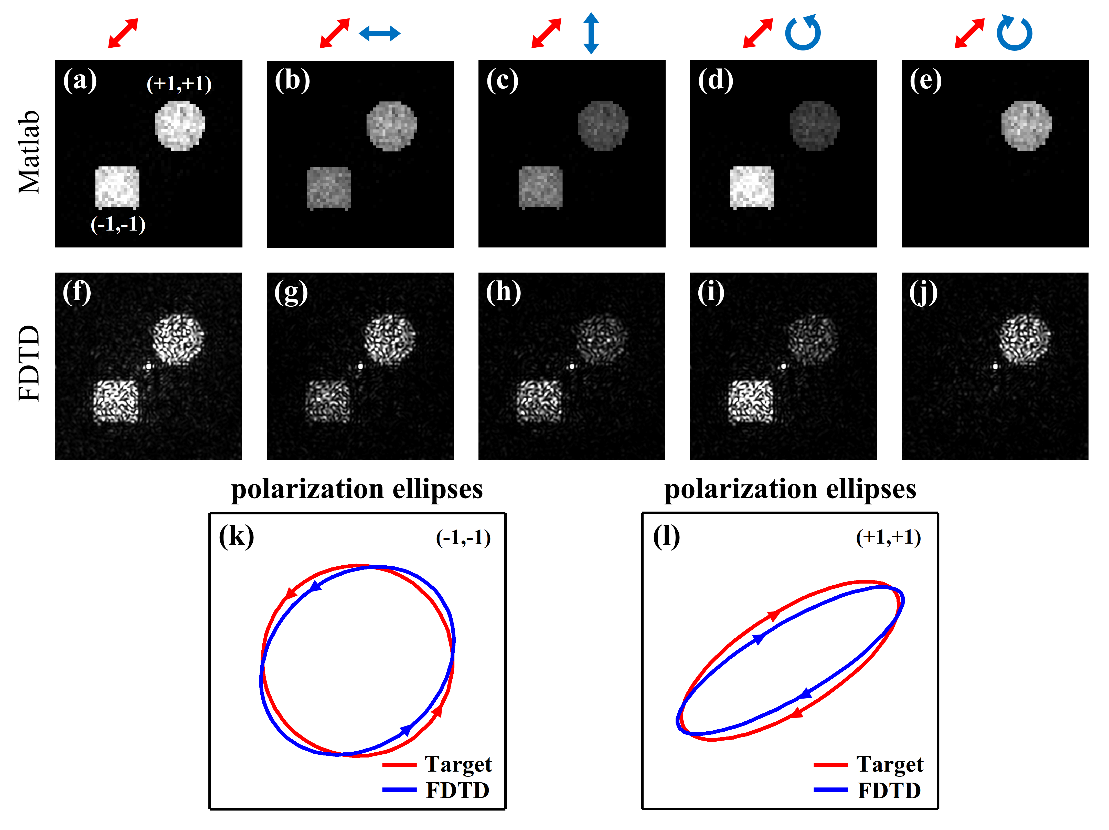
**

**Figure S1.** (a)-(j) Simulated results of the reconstructed images under different polarization analyzers. The red and blue arrows indicate the polarization of input and output light. (k)-(l) Target and simulated polarization ellipses of the reconstrcuted images in different diffraction orders.


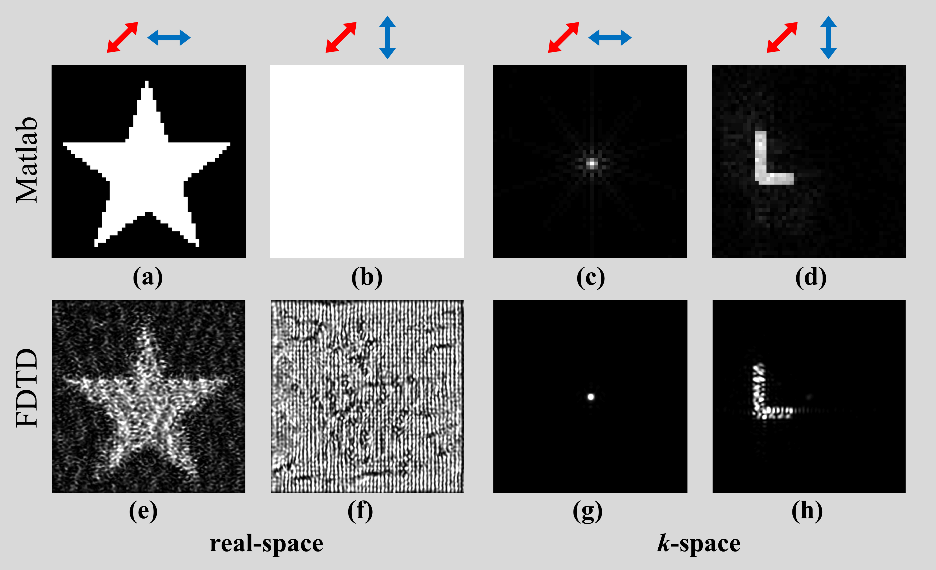


**Figure S2.** (a)-(h) Simulated results of the metasurface with the capability of realizing amplitude-only as well as phase-only modulation in *t_xx_* and *t_yy_* polarization channels, respectively. The encoded star pattern in real-space is based on amplitude modulation. Meanwhile, the reconstructed holographic image ‘L’ in *k*-space is based on the phase-only modulation.


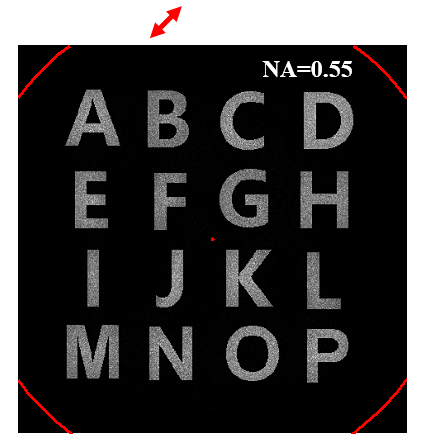


**Figure S3**. Simulated results of the reconstructed holographic images in different diffraction orders that located at the Fourier plane. The observation regions of the objective lenses with NA=0.55 is represented by the red circle.

**Table S1.** Comparison between our work and other reported vectorial holography approaches.

| **References** | **Categories of metasurfaces/working mode** | **Working wavelength (nm)** | **Vectorial holography** | **Arbitrary polarization generation** | **Diffraction orders modulation** |
| --- | --- | --- | --- | --- | --- |
| Ref. 27 (2020) | Segmented/ Transmission | 850 | √  (6 types of polarization states) | √ | Not mentioned |
| Ref. 26 (2018) | Interleaved/ Reflection | 650 ~ 850 | √  (4 types of polarization states) | √ | Not mentioned |
| Ref. 25  (2018) | Non-interleaved/ Transmission | 800 | √  (3 types of polarizations) | × | Not mentioned |
| Ref. 41  (2019) | Non-interleaved/ Transmission | 850 | √  (ultimate polarization Pattern) | √ | Not mentioned |
| Ref. 42 (2020) | Interleaved/ Transmission | 475 ~ 675 | √  (4 types of polarization states) | √ | Encoding arbitrary phase profiles+ polarization modulations (4 diffraction orders) |
| Ref. 43 (2021) | Interleaved/ Transmission | 475 ~ 675 | √  (ultimate polarization Pattern) | √ | Not mentioned |
| Our work | Non-interleaved/ Transmission | 800 | √  (16 types of polarization states) | √ | Encoding arbitrary phase profiles+ polarization modulations (16 diffraction orders) |

1. Email: huanglingling@bit.edu.cn [↑](#footnote-ref-1)
